# The relation between resident-related factors and care problems in nursing homes: a multi-level analysis

**DOI:** 10.1186/s12913-024-11915-y

**Published:** 2024-11-19

**Authors:** Suleyman Bouchmal, Yvonne M. J. Goërtz, Coen Hacking, Bjorn Winkens, Sil Aarts

**Affiliations:** 1 Living Lab in Ageing and Long-Term Care, Maastricht University, Maastricht, The Netherlands; 2 Department of Health Services Research, CAPHRI Care and Public Health Research Institute, Faculty of Health, Medicine and Life Sciences, Maastricht University, Duboisdomein 30, Maastricht, 6229 GT The Netherlands; 3 Department of Methodology and Statistics, CAPHRI Care and Public Health Research Institute, Faculty of Health, Medicine and Life Sciences, Maastricht University, Maastricht, The Netherlands

**Keywords:** Nursing homes, Quality of care, Long-term care, Care problems, Resident-related factors

## Abstract

**Background:**

Care problems such as decubitus and fall incidents are prevalent in nursing homes. Yet, research regarding explanatory factors on these care problems is scarce. The aim of this study is twofold: (1) to identify the degree to which a diverse set of resident-related factors (e.g., care dependency levels) are associated with the sum of six care problems (pressure ulcers, incontinence, malnutrition, falls, freedom restriction, and pain), and (2) to investigate which resident-related factors are associated with each of these six care problems individually.

**Methods:**

Data were collected (2016–2023) using the International Prevalence Measurements of Care Quality (LPZ). Factors such as age, number of diagnoses, and length of stay were included. While respecting nested data within eight organizations, the associations between thirteen resident-related factors and the six care problems were determined using multilevel analyses.

**Results:**

A total of 3043 residents were included (mean age 81.9; SD: 10.5). The most prevalent care problem was incontinence (*n* = 1834; 60.3%). Nurse proxy-rated confusion (*r* = 0.227; *p* < 0.001) and aggression (*r* = 0.285; *p* = 0.001) were associated strongest with the sum of the six care problems; and higher after correcting for the residents’ care dependency levels (respectively *r* = 0.504; 0.584 — both *p* < 0.001). Pre-admission risk assessments for pressure ulcers (OR 7.03), malnutrition (OR 3.57), and falls (OR 3.93) were strongest associated with individual care problems such as pressure ulcers, and falls.

**Conclusions:**

This study shows the association between several resident-related factors and care problems such as gender, years since admission, and care dependency level. Factors such as proxy-rated aggression and confusion were strongest associated with the presence of care problems, while pre-admission risk assessments were strongest associated with several individual care problems. The findings underscore the importance of prioritizing early pre-assessments, as they empower care professionals to take into account resident-specific factors and their influence on the emergence of care problems.

**Supplementary Information:**

The online version contains supplementary material available at 10.1186/s12913-024-11915-y.

## Background

As nursing homes are essential in providing long-term care for older adults, the quality of care provided in these organizations directly affects health outcomes and resident well-being [1, 2]. Several methods have been introduced to evaluate the quality of care in nursing homes [3, 4]. For example, data resources such as electronic health records (EHRs), observations by professionals or researchers using checklists, and direct information from residents through interviews or surveys are frequently used [5–9]. In addition, the prevalence of care problems such as incontinence, pressure ulcers, and falls serves as outcome measures for evaluating quality of care [10]. For example, high rates of pressure ulcers may lead organizations to consider repositioning the residents more often [11]. Researching the explanatory factors of these care problems will improve our understanding of their occurrence and development [12–14].

In the Netherlands, the International Prevalence Measurement of Care Quality program (LPZ, translated from Dutch: *Landelijke Prevalentiemeting Zorgkwaliteit*) provides insight into quality of care by measuring various explanatory factors in nursing homes [15]. Well-known explanatory factors include age and gender, as these are frequently used to predict the risk of care problems [16]. The LPZ is an annual assessment of quality in long-term care; it not only provides (inter)national organizations with insight into their quality of care but also allows for comparisons between organizations in an anonymous manner. These insights can be used as mirror data —participating healthcare organizations can compare their organization with the results of (inter)national peer organizations [17]. It combines questionnaires with data from EHRs to identify prevalent or emerging care problems. The care problems reported in the LPZ are based on their high occurrence in nursing homes and their effect on the quality of care [10, 18], including pressure ulcers, incontinence, malnutrition, falls, freedom restriction, and pain.

Although research regarding the assessment of quality of care in nursing homes is expanding, it remains challenging due to a wide variety of factors. Firstly, residents in nursing homes display a diverse set of characteristics and complex care needs, which may indicate that a one-size-fits-all approach to delivering care is not effective [19–21]. Secondly, contextual factors may influence the quality of care provided: nursing homes differ in the number of staff available, the level of nurse staffing, regulatory compliance, and facility resources [22–24]. Thirdly, various tools that assess quality of care are currently used, which hinders generalization, benchmarking, and reusability of the data gathered from various organizations [25, 26]. Therefore, little is known about the association between (a large set of) resident-related factors and the various care problems that can emerge within nursing organizations. The aim of this study is twofold: (1) to identify the degree to which a diverse set of resident-related factors (e.g., care dependency levels) are associated with the sum of six care problems (pressure ulcers, incontinence, malnutrition, falls, freedom restriction, and pain), and (2) to investigate which resident-related factors are associated with each of these six care problems individually.

## Methods

The present quantitative, explorative study stems from the International Prevalence Measurements of Care Quality (LPZ) using data collected in Dutch nursing homes from 2016 to 2023 [15].

### International prevalence measurements of care quality (LPZ 2.0)

The LPZ was developed in 1998 and directed by the Living Lab in Ageing and Long-Term Care (AWO-L, translated from Dutch*: Academische Werkplaats Ouderenzorg Limburg)* at the University of Maastricht, the Netherlands [27]. The LPZ is a multi-year, cross-sectional, multicenter, point prevalence instrument that contains assessments for healthcare organizations (e.g., hospitals and nursing homes) to analyze the quality of care [10]. This study only included nursing homes from the Netherlands.

LPZ 2.0 was introduced in 2016 and has maintained a three-part structure based on the study from Donabedian (1988). The structure focuses on measuring explanatory factors, analyzing processes based on prevention and treatments for care problems, and evaluating care outcomes using prevalence and incidence numbers [28]. The prevalence of care problem is often used as key indicators for both quality and patient safety [10]. The LPZ focuses on six highly prevalent care problems in nursing homes — pressure ulcers, incontinence, malnutrition, falls, freedom restriction, and pain — which are all indicators to express the quality of care [29]. The LPZ combines questionnaires and data from EHRs (in Dutch: *Elektronisch Cliënten Dossier* [ECD]) to investigate the provided care at three levels: (1) organization, (2) department, and (3) resident. The questionnaires include sections regarding the different levels of the organizations, the type of setting, and the outcome measures (e.g., care problems). The questionnaires on the resident level are completed on a proxy-rating basis in the presence of the resident: nurses act as a proxy to determine the indicators associated with the quality of care for every individual resident [30]. All nurses involved in the LPZ measurements, followed subsequent training on completing the LPZ questionnaires on the various care problems, with a focus on ensuring accuracy in proxy-rated assessment. Parallel to proxy-rated assessments, additional data from the resident’s EHRs was imported. One nurse entered data from bedside measurements, while another nurse confirmed the data in the EHRs. Aside from the proxy-rated assessments, no additional measurements were performed for the LPZ. Data on other resident-related factors were obtained from the EHRs.

### Resident-related factors as independent factors

This study evaluated a total of 10 factors (nine resident-level questions and the Care Dependency Scale items as a sum) in the statistical analyses: (1) date of admission; (2) having undergone an operation in the past two weeks (yes vs. no); (3) receiving end-of-life care (yes vs. no, diagnosed by an elderly care physician to shift towards palliative or terminal care); (4) care profile of the resident (known as ZZP score, Care Intensity Package, or Dutch Care Severity Package), where a higher score indicates a greater level of care requirement and more intensive and specialized care (score of 1 to 10, nominal) [31–33]; (5) proxy-reported noted confusion assessed with the question “Do you find the resident more confused lately than before?” (yes vs. no, no indicated time span); (6) proxy-reported noted aggression evaluated with the question “Has the resident exhibited any aggressive behavior in this institution in the last 7 days?” (yes vs. no). Extracted from the EHR, a pre-admission assessment is available for residents being at higher risk based on clinical judgment and registered for (7) pressure ulcers, (8) malnutrition, and (9) falls (yes vs. no). The pre-admission assessments are conducted immediately prior to admission. Utilizing clinical expertise, nurses evaluate the potential for the resident to develop specific care problems. This systematic screening process is conducted only for residents at increased risk of falls, pressure ulcers, and malnutrition, which subsequently indicates the additional pre-admission risk assessments.

The overall (10) care dependency status was assessed using the Care Dependency Scale (CDS), which is defined as a secondary need for support in the domain of care to compensate for a self-care deficit [34–37]. Trained proxy nurses conducted the assessment of residents' care dependency. The CDS is a 15-item tool, rated on a 5-point Likert scale. The total score is aggregated from the 15 items, with scores ranging from 15 to 75; a lower score indicates a higher level of care dependency. The CDS is divided into five categories: (1) sum score 15–24 indicating completely dependent, (2) sum score 25–44 indicating dependent to a great extent, (3) sum score 45–59 indicating partially dependent, (4) sum score 60–69 indicating independent to a great extent, and (5) sum score 70–75 indicating completely independent [38]. In the present study, care dependency was considered continuous for both aims, expressed as the sum score ranging from 15–75, based on earlier studies [35–37]. Nursing homes evaluate the CDS annually or following significant life events (e.g., falls). The most recent CDS sum score were extracted from the EHRs and used for the assessment on the measurement day.

### Care problems as dependent factors

Regarding the first research aim, the study identifies the degree to which resident-related factors in nursing homes located in the Netherlands are associated with the present care problems. For the second aim, the study explores which of the resident-related factors were associated with each specific care problem for residents in nursing homes. The analysis evaluated the care problems separately. The combination of present care problems (0–6) was the outcome measure and was identified by the LPZ. The following care problems were assessed: (1) pressure ulcers, (2) incontinence, (3) malnutrition, (4) falls, (5) freedom restriction, and (6) pain.

#### Pressure ulcers

Risk assessment for pressure ulcers was conducted based on the Braden assessment tool (sum score 6–23) [29, 39]. The presence of the care problem was defined as pressure ulcer category 1 or higher, meaning that at least one affected spot on the resident’s body was observed.

#### Incontinence

Urine incontinence was assessed by the presence or absence of this care problem; it is defined as any form of involuntary urine loss [40]. In the LPZ, urinary incontinence diagnoses are only used for residents without catheters and may occur due to stress, high urge, overflow, or functional independencies—combinations are possible [29].

#### Malnutrition

Residents are malnourished if they meet one of the two following criteria: (1) body mass index (BMI) < 18.5 (resident age 65 ≥ is BMI ≤ 20.0) or (2) unintentional weight loss of more than 6 kg in the last 6 months or more than 3 kg in the last month [29].

#### Falls

The LPZ maintains two definitions, one based on measuring the incidence of falls and the second describing falls. (1) Incident falls: the incidence represents the number of residents who fell one or more times (with or without injuries) during a given period. The incidence measurement of falls covers 30 days prior to the measurement day, and (2) the definition of a fall is based on the definition of the Kellogg International Work Group (1987), which states that a fall is an event in which the resident unintentionally lands on the ground or a lower level [41]. Falls were included if they occurred and were documented within 30 days prior to the measurement day. If it is unknown whether the resident had an incidence of falling, this was considered as an absence of the care problem.

#### Freedom restriction

Freedom restriction and associated measures are divided by two definitions in the LPZ. (1) Incident restriction measures are the number of residents for whom a restraint measure was applied during a given period. In the LPZ, the incidence measurement of restraint measures was measured 30 days prior to the measurement day. (2) Restraints, often referred to as protective measures, aim to prevent dangerous or high-risk situations or to enable medical treatment [29]. The application of a measure that cannot be lifted independently, such as a deep chair or a tabletop, is recorded in the LPZ as a freedom-restricting measure, regardless of whether the resident had provided consent and regardless of the purpose of applying these measures.

#### Pain

The LPZ follows the definition from the Association for the Study of Pain (IASP), describing pain as “An unpleasant, sensory, and emotional experience associated with actual or potential tissue damage or described in terms of such damage” [42]. Pain was assessed by the presence of pain on two levels: (1) pain on a daily basis and (2) pain but not on a daily basis, both evaluated for the last 7 days. In this study, both levels were considered as the care problem being present. In the LPZ, pain was evaluated through proxy assessment.

### Potential confounders

Several potential confounders were considered: age (in years), gender (male or female), and the number of diagnosis based on the International Classification of Diseases and Related Health Problems (ICD-10) with a predefined list of 27 problems varying from somatic to cognitive diseases, e.g., endocrine diseases to psychological or behavioral disorders (see Appendix 1: score 0 to 27) [43] (i.e., one person could have more than one [chronic] medical diagnosis at the time of assessment).

### Statistical analysis

Data preprocessing was conducted in SPSS by removing duplicates and cleaning the unbalanced measurements, confirming that this study is only left with single, unique cases [44]. Due to the use of data without unique identifiers for each resident, this study removed duplicates and cleaned unbalanced measurements based on factors such as age, gender, years since admission, CDS level, ZZP score, and the total number of care problems. However, since the data spanned from 2016 to 2023, all factors except gender could have changed over time. This suggests that some repeated measurements may have been included but not accounted for in the analysis. Imputation for missing values was considered, but due to the low substantive consistency between independent factors and the number of missing assessments for residents within the organizations, imputation was not performed [45]. In total, 766 duplicates were removed, resulting in 3,043 cases included in this study.

The present study covered a two-level nested structure, where all residents were nested within one of the eight organizations. The data were analyzed using linear mixed models (LMMs) and generalized logistic mixed models (GLMMs) in Python, which both comply with a multilevel approach, respecting the hierarchical structure of (longitudinal) data sets [46–48]. Multilevel models for both aims consisted of fixed terms, which included all independent factors and the random effect—not the observable effect—for the residents. Here, the random effect was the different organizations in which the residents are nested (e.g., contextual factors). As no interaction effects were assumed, no random slopes were included for the independent factors [49].

#### First aim – analysis on sum of care problems

Two models were conducted for analyzing the regression coefficients and their *p*-value for all factors possibly associated with the sum of care problems. Before the analyses were conducted, multicollinearity was checked using the variance inflation factor (VIF). As the literature varies regarding threshold values for determining collinearity, this study follows an earlier study conducted by Everink et al. (2021) that used also an LPZ dataset for analyzing a specific care problem in nursing homes. Here, a VIF value ≤ 9 was considered acceptable [16]. No multicollinearity was identified in the present study. The underlying items of the CDS were not checked for multicollinearity regarding the care problems, so the mutual relationship of the other independent factors is reported by excluding the CDS sum score. The first model included all factors regarding the sum of the care problems. The second model excluded the CDS sum score as an independent factor, as the present study has no overview of the score per item but only the sum score. Previous research concluded that several items and their scores within the CDS could explain significant differences between individuals [50]. The ZZP score was excluded for here due to the organizations’ inconsistency and uneven distribution in the provided categories. Moreover, it was expected that several care problems are overrepresented in higher ZZP scores.

#### Second aim – analysis for individual care problems

The independent factors were related to the six care problems individually. All factors were analyzed in six separate GLMMs, each addressing one care problem; corresponding odds ratios, 95% confidence interval, and associated *p*-values were reported. All outcomes were considered statistically significant by *p*-values of 0.05 (2-tailed) or less [51]. In addition, the interclass correlation coefficient (ICC) was calculated for assessing the reliability of measurements by multiple testing and was reported for each model [52]. An ICC-score (0–1) indicates how much of the total variability in the outcome measurement is due to the differences between nursing homes – a higher number is indicative for a higher correlation between nursing homes organizations on the outcome measures [53]. Research lacks formal thresholds, but lower ICC-scores suggest that the variability if primarily due to differences among resident themselves – rather than being caused by the correlation between outcomes measures (e.g., care problems) and the nursing homes [54]. This study provides the first steps in multilevel analysis regarding resident-related factors with an exploratory approach, in which there was no need for adjustments for multiple testing.

### Ethical considerations

The Medical Research Ethics Committee of Maastricht University and Maastricht University Medical Centre approved the LPZ study. All nursing homes that were included in the LPZ study participated on a strictly voluntary basis, with no financial compensation [10]. Each resident was informed of the study and the measurements, and each resident or their representative had to give informed verbal consent. All data were collected and processed anonymously.

## Results

The descriptive characteristics are reported in Table 1. The residents were unevenly distributed within the nested structure of eight nursing homes (groups ranged from 73 to 1,035), and the majority were female (65.2%) and had three or more diagnoses (57.6%). Furthermore, the years since admission (mean = 4.99; SD = 3.27) were reported, and the CDS sum score of 25–44 (category 2) describing a level of dependence to a great extent was most present (*n* = 962, 31.6%). Of the ZZP categories (range 1–10), category 4 was the most present (*n* = 1,429, 46.9%). Most residents had pre-admission risk assessments available in their EHRs: pressure ulcers (*n* = 1,865, 61.3%), malnutrition (*n* = 1,676, 55.1%), and falls (*n* = 1,908, 62.7%). In addition, residents who received end-of-life treatment (*n* = 297, 9.8%) and who had undergone surgery in the previous two weeks (*n* = 26, 0.9%) are reported. The condition of residents’ behavior was reported based on a proxy rating, checking for confusion (*n* = 416, 13.7%) and aggression (*n* = 231, 7.6%).

**Table 1:** Descriptive characteristics of the study population (*N* = 3043)

Characteristics	
Mean age in years (SD)	81.9 (10.2)
Median years since admission (IQR)	4 (3—6)
Female (%)	1,985 (65.2)
Median sum of care problems (IQR)	2 (0 – 4)
Prevalence of care problems (%)	
Pressure Ulcers	309 (10.2)
Incontinence	1,834 (60.3)
Malnutrition	460 (15.1)
Falls	379 (12.5)
Freedom restrains	980 (32.2)
Pain	1,108 (36.4)
Number of medical diagnoses (%)	
0	24 (0.8)
1	502 (16.5)
2	765 (25.1)
3 ≥	1,752 (57.6)
Care Profile ZZP-categories (%)^a^	
1	0 (0.0)
2	0 (0.0)
3	30 (1.0)
4	419 (13.8)
5	1,429 (46,9)
6	787 (25.9)
7	180 (5.9)
8	13 (0.4)
9	34 (1.1)
10	3 (0.1)
Missing	148 (4.9)
CDS-categories (%)^b^	
1	348 (11.4)
2	962 (31.6)
3	924 (30.4)
4	518 (17.0)
5	291 (9.6)
Risk assessment available in health record:	
Pressure Ulcer (% Yes)	1,865 (61.3)
Falling (% Yes)	1,908 (62.7)
Malnutrition (% Yes)	1,676 (55.1)
Receiving end-of-life care (% Yes)	297 (9.8)
Received operation in past two weeks (% Yes)	26 (0.9)
Noted confusion (% Yes)	416 (13.7)
Noted aggression (% Yes)	229 (7.6)

^a^ZZP-categories (3 to 10) describe care profile, the indication not officially used since 2015 in the Netherlands

^b^CDS-categories for care dependency (1) sum score 15-24 completely dependent, (2) sum score 25-44 dependent to a great extent, (3) sum score 45-59 partially dependent, (4) sum score 60-69 independent to a great extent, and (5) sum score 70-75 completely independent

In total, 82.4% (*n* = 2,509) of the residents had at least one care problem (see Table 1). The most frequently reported care problem was incontinence (*n* = 1,834, 60.3%), followed by pain (*n* = 1,108, 36.4%). The care problem with the lowest frequency was pressure ulcers (*n* = 309, 10.2%). Across all eight nursing homes, 82% to 85% of residents had at least one care problem, indicating that 15% to 18% of residents did not experience any care problems. Regarding the prevalence of individual care problems in 2016–2023 in the nursing homes: pressure ulcers (range 3 to 138, mean = 39), incontinence (range 45 to 609, mean = 229), malnutrition (range 11 to 156, mean = 58), falls (range 7 to 116, mean = 47), freedom restrains (range 17 to 423, mean = 123), and pain (range 25 to 369, mean = 139).

### Association between resident-related factors and the sum of care problems

Eight resident-related factors were found to be associated with the care problems in a statistically significant way (see Table 2): gender, years since admission, having undergone surgery, number of diagnoses, CDS sum score, the present risk assessment for pressure ulcers, noted confusion, and aggression. The proxy-rated noted aggression of the residents’ behavior showed the strongest (positive) regression coefficient of 0.285 (SE = 0.089; t = 3.212; *p* < 0.001).

**Table 2:** Multilevel analyses for the association between resident-related factors and care problems – before and after CDS sum score correction

		Present care problems		
	Parameter estimate	*P*-value	Parameter estimate	*P*-value
Age	−0.001	0.988	−0.001	0.565
Gender	0.193	**< 0.001**	0.231	**< 0.001**
Years since admission	−0.024	**0.001**	−0.017	**0.030**
Undergone surgery	0.157	0.529	0.135	0.613
End-of-life care	−0.249	**0.001**	-.0249	**0.003**
Number of diagnoses	0.079-	**< 0.001**	0.080	**< 0.001**
CDS sum score	0.031	**< 0.001**		
Risk assessment Pressure Ulcers	0.250	**< 0.001**	0.359	** < 0.001**
Risk assessment Malnutrition	−0.015	0.818	−0.001	0.999
Risk assessment Falls	−0.012	0.850	−0.057	0.399
Noted confusion	0.277	**< 0.001**	0.504	**< 0.001**
Noted aggression	0.285	**0.001**	0.584	**< 0.001**
Organization ICC	0.194		0.218	

Coefficient is significant at the .05 level (2-tailed)

The CDS sum score was excluded in the second model as an independent factor for the first aim (Table 2). The CDS sum score (a higher score is indicative of a lower level of dependency) aggregates 15 items, some of which are directly linked to specific care problems. After correcting for the CDS sum score, noted aggression was again the strongest (positive) regression coefficient, at 0.584 (SE = 0.094; t = 6.209; *p* < 0.001). Here, the factors of gender, years since admission, end-of-life care, number of diagnoses, risk assessment of pressure ulcers, and noted aggression and confusion were associated with the present care problems in a statistically significant way, although the regression weights were small. The associations between the factors and all six care problems were reported (Appendix 2b).

### Association between resident-related factors and the individual care problems

Regarding the second aim, the present study investigates the association between resident-related factors and each care problem separately (Table 3). Regarding the first care problem, pressure ulcers, four factors were significantly related: number of diagnoses, CDS sum score, present pre-admission risk assessment for pressure ulcers, and present pre-admission risk assessments for falling. The strongest (positive) association regarding pressure ulcers was found in the risk assessment for pressure ulcers, with an odds ratio (OR) of 7.03 (95% CI 4.859–10.166; *p* < 0.001).

**Table 3:** Multilevel analyses for the association between resident-related factors and care problems individually

	Presence of Pressure Ulcers			Presence of Incontinence			Presence of Malnutrition			Presence of Falling			Presence of Freedom Restrains			Presence of Pain		
	Odds Ratio	95% CI	*P*-value	Odds Ratio	95% CI	*P*-value	Odds Ratio	95% CI	*P*-value	Odds Ratio	95% CI	*P*-value	Odds Ratio	95% CI	*P*-value	Odds Ratio	95% CI	*P*-value
Age	0.998	L0.985 U1.011	0.811	1.006	L0.997 U1.015	0.180	1.022	L1.010 U1.035	**0.001**	1.012	L0.999 U1.025	0.072	1.007	L0.998 U1.016	0.123	0.994	L0.956 U1.002	0.163
Gender	0.784	L0.601 U1.022	0.073	1.306	L1.091 U1.562	**0.004**	1.960	L1.538 U2.501	**< 0.001**	0.781	L0.617 U0.989	**0.041**	0.886	L0.739 U1.063	0.193	1.404	L1.191 U1.656	**< 0.001**
Years since admission	1.012	L0.972 U1.054	0.570	1.068	L1.037 U1.100	**< 0.001**	0.939	L0.904 U0.976	**0.001**	0.894	L0.852 U0.938	**< 0.001**	0.920	L0.892 U0.949	**< 0.001**	0.983	L0.959 U1.008	0.168
Undergone surgery	1.864	L0.637 U5.467	0.256	0.653	L0.266 U1.604	0.354	0.975	L0.274 U3.464	0.968	1.711	L0.557 U5.258	0.349	0.995	L0.388 U2.554	0.993	1.070	L0.473 U2.421	0.871
End-of-life care	1.156	L0.630 U2.120	0.638	1.211	L0.819 U1.790	0.336	0.971	L0.636 U1.482	0.892	1.033	L0.635 U1.680	0.895	1.041	L0.798 U1.530	0.838	0.975	L0.705 U1.347	0.877
Number of diagnoses	1.135	L1.126 U1.144	**< 0.001**	0.999	L0.949 U1.052	0.969	0.993	L0.933 U1.057	0.826	1.058	L0.991 U1.130	0.095	1.027	L0.976 U1.081	0.301	1.182	L1.129 U1.237	**< 0.001**
CDS sum score	0.969	L0.961 U0.977	**< 0.001**	0.928	L0.922 U0.934	**< 0.001**	0.979	L0.973 U0.986	**< 0.001**	0.987	L0.980 U0.994	**< 0.001**	0.952	L0.947 U0.957	**< 0.001**	0.997	L0.992 U1.002	0.334
ZZP score	0.996	L0.990 U1.002	0.168	0.997	L0.992 U1.002	0.182	0.999	L0.993 U1.006	0.670	1.001	L0.994 U1.008	0.788	1.008	L1.003 U1.013	**0.001**	1.001	L0.997 U1.005	0.540
Risk assessment Pressure Ulcer	7.028	L4.859 U10.166	**< 0.001**	1.308	L1.017 U1.683	**0.037**	0.667	L0.491 U0.906	**0.009**	0.487	L0.350 U0.677	**< 0.001**	1.172	L0.917 U1.498	0.204	1.302	L1.047 U1.619	**0.018**
Risk assessment Malnutrition	0.803	L0.560 U1.152	0.236	0.780	L0.604 U1007	0.056	3.574	L2.622 U4.871	**< 0.001**	0.833	L0.602 U1.153	0.270	0.838	L0.653 U1.076	0.165	0.893	L0.715 U1.116	0.321
Risk assessment Falls	0.413	L0.286 U0.596	**< 0.001**	1.032	L0.806 U1.321	0.800	0.811	L0.599 U1.099	0.178	3.934	L2.859 U5.413	**< 0.001**	1.824	L1.431 U2.324	**< 0.001**	0.942	L0.769 U1.169	0.588
Noted confusion	0.742	L0.524 U1.130	0.093	0.859	L0.664 U1.111	0.246	1.152	L0.867 U1.530	0.329	1.838	L1.386 U2.438	**< 0.001**	1.527	L1.203 U1.938	**< 0.001**	1.420	L1.140 U1.769	**0.002**
Noted aggression	0.721	L0.446 U1.165	0.184	0.820	L0.581 U1.158	0.259	1.370	L0.956 U1.964	0.086	1.822	L1.283 U2.587	**< 0.001**	2.034	L1.493 U2.771	**< 0.001**	1.162	L0.871 U1.551	0.307
Organization ICC		0.134			0.057			0.038			0.025			0.093			0.014	

*95% CI* 95% Confidence Interval for Odds Ratio, *L* Lower bound, *U* Upper bound, *CDS* Care Dependency Scale, *ZZP* Care profile of residents in Dutch nursing homes

Regarding the second care problem, incontinence, four factors were significantly associated: gender, years since admission, CDS sum score, and present pre-admission risk assessment for pressure ulcers. The strongest (positive) association was found in the pre-admission risk assessment for pressure ulcers: an OR of 1.31 (95% CI 1.017–1.683; *p* = 0.037).

Third, regarding the presence of malnutrition, six factors were significant: age, gender, years since admission, CDS sum score, and present pre-admission risk assessments for pressure ulcers and malnutrition. The strongest (positive) associated factor was the risk assessment for malnutrition: an OR of 3.57 (95% CI 2.622–4.871; *p* < 0.001).

Fourth, seven factors were statistically significant regarding the presence of falling based on the regression coefficient: gender, years since admission, CDS sum score, present pre-admission risk assessment of pressure ulcers and falls, and noted aggression and confusion. The strongest (positive) association was found with the risk assessment for falling: an OR of 3.93 (95% CI 2.859–5.413; *p* < 0.001).

Fifth, regarding the presence of freedom restraints, six factors were significantly associated: years since admission, CDS sum score, present pre-admission risk assessments for falling, and noted confusion and aggression. The strongest (positive) association was found by the factor of noted aggression: an OR of 2.03 (95% CI 1.493–2.771; *p* < 0.001).

Lastly, regarding the sixth care problem, four factors were statistically associated with pain: gender, number of diagnoses, present pre-admission risk assessment for pressure ulcers, and noted confusion. The strongest (positive) association was found in the factor of noted confusion: an OR of 1.42 (95% CI 1.140–1.769; *p* = 0.002). In addition, the associations between all resident-related factors and care problems individually were reported (Appendix 2. c).

## Discussion

The aim of this study is twofold: (1) to identify the degree to which a diverse set of resident-related factors (e.g., care dependency levels) are associated with the sum of six care problems (pressure ulcers, incontinence, malnutrition, falls, freedom restriction, and pain), and (2) to investigate which resident-related factors are associated with each of these six care problems individually. Of the six care problems, incontinence was found to be the most prevalent care problem, followed by pain. Moreover, proxy-noted confusion and aggression showed the strongest association with the six care problems. The pre-admission risk assessment for pressure ulcers, malnutrition, and falls showed the strongest association with the corresponding care problems.

Regarding the prevalence rates in nursing homes, comparable findings for incontinence (60.3%) were found in earlier studies [55–57], as well as the association between the care problem and being female [58–60]. The second most common care problem, pain (36.4%), is the most difficult to measure in practice (e.g., difficult to observe, behavior disturbance)—especially in people with dementia [61, 62]. A recent study found comparable results regarding prevalence while analyzing the pain of nursing home residents in Austria in 2016–2019, concluding that 40.4% (*n* = 1,239) of the residents in nursing homes experienced pain [63]. In addition, the results of the present study indicate that females experience more pain than men do, which is in congruence with previous studies [64–66]. Interestingly, the prevalence of freedom restraints (the third most prevalent care problem) in nursing homes is still high, even after introducing the 2020 Care and Coercion Act (*Wet Zorg en Dwang)* in the Netherlands [67]. This act should reduce the use of coercion or restraints in healthcare unless a strict plan is made in collaboration with the residents and their loved ones, and they may only be used as the last option.

When reviewing the factors associated to individual care problems, it becomes apparent that CDS sum score, number of years since admission, gender, and pre-admission risk assessment for pressure ulcers are factors that are often significantly associated with the presence of individual care problems. Firstly, several studies identified thresholds based on the CDS sum score for residents being at risk for individual care problems [68, 69]. By analyzing the specific 15 items within the scale, the literature shows that the CDS identifies factors that are related to the care problems of pressure ulcers, malnutrition, falling, and incontinence [35, 50, 70–73]. Moreover, the level of dependency is often an explanatory factor for the use of restrains [74–76]. Secondly, regarding the number of years since a resident’s admission, it remains challenging to investigate associations, as age increases accordingly. However, the present results showed that number of years since admission was associated with more individual care problems than the age of a resident. Thirdly, being female is significantly associated with more presence of incontinence [55–59], pain [64–66], and malnutrition [16, 77, 78]. However, males appeared to have higher odds for the presence of falling, indicating that men are more likely to fall. In the literature, the association between gender and falling is often not strongly associated or consistent when assessed in nursing [79–81]. Lastly, pre-admission risk assessment for pressure ulcers indicated a higher likelihood of the care problems of pressure ulcers, incontinence, and pain, but it was negatively associated with malnutrition and falling. The pre-admission risk assessment does not differentiate between being at risk and already having pressure ulcers. For both pressure ulcers and incontinence, this could indicate that the care problems may already be present pre-admission. The presence of the care problem of pain was assessed in the last 7 days. It is unknown if residents develop multiple care problems, as a resident may develop pain due to earlier pressure ulcers [82].

Confusion and aggression appeared to be strongly associated with the sum of all care problems. Previous research already discovered these factors as predictors of care problems—in particular, the use of restraints in nursing homes [83–86]. Furthermore, confusion has been identified as an explanatory factor in previous studies regarding the presence of pain [87, 88]. Other studies concluded a reversed causation, stating that the use of restraints may lead to a state of confusion [89]. Interestingly, when excluding the CDS sum score as an explanatory factor within the first aim, the regression coefficients in the first aim of the factors of confusion and aggression increased prominently. This may indicate that both confusion and aggression are highly associated with specific items within the CDS, which is in line with previous studies [90]. Moreover, aside from aggression and confusion, other items in the CDS that may indicate a higher risk of falling (e.g., low mobility) are often reasons for restraining residents (e.g., to prevent falling), as the pre-admission risk assessment for falling is significantly associated with the presence of freedom restrains [91, 92].

To our knowledge, this study is the first to provide an overview of a diverse set of resident-related factors within as many as eight long-term care organizations, thereby providing a clear state-of-the-art overview of the prevalence of these care problems and their associated factors. By conducting the analysis using linear mixed models and generalized linear mixed models, the nested structure was respected, as residents are prone to experiencing contextual factors within the organizations they reside in. However, this study also needs to be considered in light of some possible limitations. Firstly, the LPZ measures and gathers data based on a cross-sectional method, which only allows for analyzing associations and not cause-and-effect relationships [93]. Furthermore, as this study serves as explorative research, an alpha level of 0.05 was approved; corrections for multiple testing should be in place in less exploratory studies (e.g., Bonferroni or Holm) [94]. Secondly, this study did not differentiate between the 15 items that are included within the CDS or the 27 included diagnoses, as the specific items or diagnoses may be strongly associated with certain care problems. Including the number of diagnoses in this study may concealed certain associations between specific diagnoses and the development of care problems. By differentiating between the CDS items and the included diagnoses, the interpretation of the results becomes more reliable. Moreover, by including all items and diagnoses separately, checking for multicollinearity becomes more justified here [95]. Thirdly, this study considered all residents as unique cases, while repeated measurements could occur in the longitudinal data (2016–2023). As no unique identifier was included in the dataset, this study could not correct the repeated measurements. This study investigated this by conducting a sensitivity analysis only including residents’ data from 2023. Therefore, the factor for years since admission was excluded, as this was considered constant. The sensitivity analysis was performed to assess the robustness of our methods given that several assumptions were made, such as the choice of included variables and random effects for both models [96]. This analysis aimed to ensure that the results were not solely influenced by specific assumptions or decisions, such as the selection of fixed effects (resident-related factors), the assumption of normal distribution of all variables based on the central limit theorem, and the assumption of no interaction effects. To reduce uncertainty related to the assumptions made in the methodology, an additional model was conducted using only the 2023 cohort, allowing for comparison of the results. The findings showed no significant differences in the outcomes, indicating that the modelling process and results in this study were appropriate [97].

Future studies on the associations between resident-related factors and care problems might be differentiating in the set of included factors. Examining the influence of various (chronic) diseases on the development of care problems by differentiating between the items in the CDS: insights may provide a better understanding of mutual relationships between specific diagnoses and residents’ care dependency levels. This allows for mapping the progress from disease to emerging care problems in nursing homes based on the change in care dependency levels. In addition, more longitudinal studies, doing justice to the development of these explanatory factors and care problems in time, should be deployed.

The findings of this study underscore the importance of prioritizing early pre-assessments, as they empower care professionals to take into account resident-related factors and their influence on the emergence of care problems. While pre-admission risk assessments are important, ongoing evaluations during a resident's stay can offer crucial insights, as care issues may develop over time due to different pre-admission circumstances (e.g., emergency or planned admissions). Continuous monitoring of risk is vital, particularly following significant life events or in response to age-related decline. However, care professionals face challenges in effectively utilizing data-informed care, underscoring the necessity for collaborative, multidisciplinary approaches that embrace concepts such as positive health and prevention. By concentrating on preventive strategies, care professionals can proactively address possible care problems, resulting in more timely interventions and an enhanced quality of care for residents in nursing homes [98, 99].

## Conclusion

This study provides evidence for associations between a diverse set of resident-related factors and highly prevalent care problems within nursing homes. Noteworthy factors that were highly associated with the sum of all care problems were the proxy-rated confusion and aggression of residents. Furthermore, when analyzing the individual care problems separately, the pre-admission risk assessments for pressure ulcers, malnutrition, and falls were the strongest explanatory factors for several care problems including pressure ulcers, incontinence, malnutrition, and falls. The care dependency sum score was negatively associated with all care problems. These insights will give direction for a more comprehensive approach for healthcare workers to consider resident-related factors and their influence on care problems, focusing on prevention to improve the quality of care for older people in nursing homes.

## Supplementary Information

Supplementary Material 1.

Supplementary Material 2.

## Data Availability

The datasets analyzed during the current study are provided by the International Prevalence Measurement of Care Quality program (LPZ), but restrictions apply to the availability of the data, which were used under license for the current study, and so are not publicly available. Data are however available from the authors upon reasonable request and with permission of LPZ.
