# Supplementary material for: The relation between resident-related factors and care problems in nursing homes: a multi-level analysis
Source: BMC Health Serv Res. 2024 Nov 19;24:1435. doi: 10.1186/s12913-024-11915-y (PMC11577899; doi:10.1186/s12913-024-11915-y)
Supplement: Supplementary file 1 — Supplementary Material 1. [file 12913_2024_11915_MOESM1_ESM.pdf]

Appendix I

Predefined list by the LPZ of types of diagnosis based on the International Classification of Diseases and Related Health Problems (ICD-10).

- Infectious diseases and parasitic diseases
- Newforming - Cancer/neoplasm
- Diseases of blood or blood-forming organs and disorders of the immune system
- Endocrine diseases and nutritional- and metabolic disorders, except diabetes mellitus
- Diabetes mellitus
- Mental and behavioral disorders, except dementia and addictions
- Dementia
- Addictions
- Diseases of the nervous system, except spinal cord injury and paraplegia
- Spinal cord injury and paraplegia
- Diseases of the eye and adnexa
- Diseases of the ear and mastoid processes
- Diseases of the cardiovascular system, expect Cerebro Vascular Accident (CVA)
- CVA
- Diseases of the respiratory system
- Diseases of the digestive system
- Diseases of the skin and subcutis
- Diseases of the bony musculature and connective tissue
- Diseases of the genitourinary system
- Pregnancy, childbirth, and the puerperium
- Congenital defects, malformations, and chromosomal abnormalities
- Symptoms, abnormal clinical findings, and laboratory results - not elsewhere classified
- Injury, poisoning, and certain other effects of external causes, expect drug overdose
- Drug overdose
- External causes of morbidity or mortality
- Factors affecting health status and contact with healthcare
- None/unknown.
