# Supplementary material for: The relation between resident-related factors and care problems in nursing homes: a multi-level analysis
Source: BMC Health Serv Res. 2024 Nov 19;24:1435. doi: 10.1186/s12913-024-11915-y (PMC11577899; doi:10.1186/s12913-024-11915-y)
Supplement: Supplementary file 2 — Supplementary Material 2. [file 12913_2024_11915_MOESM2_ESM.pdf]

Appendix II

Table A: Testing for Multicollinearity

	Collinearity Statistics	
	Tolerance	VIF
Age	0.875	1.142
Gender	0.952	1.051
Years since admission	0.953	1.049
Undergone surgery	0.995	1.005
End-of-life care	0.966	1.035
Number of diagnoses	0.968	1.033
CDS sum score	0.935	1.069
Risk assessment Pressure Ulcers	0.505	1.980
Risk assessment Malnutrition	0.483	2.072
Risk assessment Falls	0.546	1.832
Noted confusion	0.953	1.049
Noted aggression	0.947	1.056

Table B: Correlation between independent factors and care problems

	Present Care Problems	
	Pearson Correlation	P-value
Age	0.006	0.752
Gender	0.065	**<0.001**
Years since admission	-0.014	0.445
Undergone surgery	0.003	0.861
End-of-life care	-0.033	0.068
Numbers of diagnosis	0.103	**<0.001**
CDS sum score	-0.395	**<0.001**
ZZP	0.003	0.867
Risk assessment Pressure Ulcers	0.108	**<0.001**
Risk assessment Malnutrition	0.064	**<0.001**
Risk assessment Falls	0.056	**0.002**
Noted confusion	0.134	**<0.001**
Noted aggression	0.122	**<0.001**

Correlation is significant at the .05 level (2-tailed)

Table C: Correlation between independent factors and care problems individually

	Pressure Ulcers		Incontinence		Malnutrition		Fall		Freedom Restraints		Pain	
	Pearson Corr.	P-Value	Pearson Corr.	P-Value	Pearson Corr.	P-Value	Pearson Corr.	P-Value	Pearson Corr.	P-Value	Pearson Corr.	P-Value
Age	-0.059	**0.001**	0.031	0.083	0.084	**<0.001**	.0048	**0.009**	0.031	0.092	-0.031	0.083
Years since admission	0.004	0.818	0.104	**<0.001**	-0.034	0.059	-0.085	**<0.001**	-0.041	**0.023**	-0.027	0.136
Gender	-0.042	**0.019**	0.066	**<0.001**	0.108	**<0.001**	-0.036	**0.047**	-0.015	0.403	0.058	**0.001**
Undergone surgery	0.028	0.128	-0.019	0.283	-0.009	0.609	0.008	0.650	-0.010	0.563	0.004	0.827
Diagnosis	0.067	**<0.001**	-0.007	0.698	-0.001	0.960	0.029	0.114	-0.029	0.112	0.134	**<0.001**
CDS	-0.173	**<0.001**	-0.472	**<0.001**	-0.128	**<0.001**	-0.089	**<0.001**	-0.334	**<0.001**	-0.047	**0.009**
End-of-life	-0.045	**0.014**	-0.045	**0.013**	0.019	0.298	-0.007	0.713	-0.020	0.258	-0.021	0.246
ZZP	0.052	**0.004**	-0.048	**0.008**	-0.036	**0.048**	-0.027	0.130	0.004	0.840	0.035	0.051
Risk assess. PU	0.140	**0.001**	0.063	**<0.001**	0.032	0.076	-0.041	**0.022**	0.090	**<0.001**	0.024	0.191
Risk assess. Mal.	-0.007	0.701	0.034	0.064	0.134	**<0.001**	-0.001	0.935	0.092	**<0.001**	-0.025	0.167
Risk assess. Fall.	-0.074	**0.001**	0.039	**0.032**	0.050	**0.005**	0.112	**<0.001**	0.111	**<0.001**	-0.024	0.293
Confusion	0.037	**0.040**	0.065	**<0.001**	0.048	**0.008**	0.116	**<0.001**	0.117	**<0.001**	0.065	**<0.001**
Aggression	0.002	0.902	0.063	**0.001**	0.049	**0.007**	0.102	**<0.001**	0.134	**<0.001**	0.031	0.091

Correlation is significant at the .05 level (2-tailed) PU= Pressure Ulcers, Mal= Malnutrition, Fall= Falls
